# The impact of Sudan armed conflict and coping strategies on the mental health of the older adult internally displaced persons in Darfur camps

**DOI:** 10.1186/s40359-024-02214-6

**Published:** 2025-02-04

**Authors:** Saif A. Musa, Abdalla A. R. M. Hamid

**Affiliations:** 1 Department of Psychology, College of Education, Sudan University of Science and Technology, Khartoum, Sudan; 2 Department of Clinical Psychology, College of Medicine & Health Sciences, United Arab Emirates University, Al Ain, United Arab Emirates

**Keywords:** Armed connflict, Distress, Coping strategies, Older adult IDPs, Darfur

## Abstract

**Background:**

The war victims in Darfur witnessed the killing of relatives, rape of women, and loss of property. Studies in war-affected populations have reported high rates of mental health problems. The present study aimed at investigating the effects of the armed conflict in Sudan on the mental health of the older adult IDPs in Darfur as well as the role of coping strategies in dealing with psychological distress.

**Methods:**

A purposive sampling technique was used to select 109 older adult internally displaced persons (*M*age = 74.93; SD = 7.07). The General Health Questionnaire (GHQ 28), the Coping Inventory for Stressful Situations (CISS), and a demographic survey were used to collect data.

**Results:**

The results revealed a high prevalence (75.2%) of non-psychotic psychiatric disorders amongst the older adult IDP participants. Task-focused coping was negatively related to somatic symptoms (*p* < 0.01), anxiety (*p* < 0.05), and general distress (*p* < 0.01), while avoidance was negatively related to age (*p* < 0.05) and social dysfunction (*p* < 0.05). Family size was positively associated with task-focused coping (*p* < 0.05) and negatively associated with somatic symptoms (*p* < 0.05). The results further showed significant gender differences in somatic symptoms (*p* < 0.001), anxiety (*p* < 0.05), general distress (*p* < 0.01), task-focused (*p* < 0.001) and avoidance coping (*p* < 0.05).

**Conclusion:**

The findings of this study suggest that attention should be directed towards fostering positive coping capacity and the provision of psychosocial support services for older adult IDPs in Darfur.

## Introduction

Armed conflict represents one of the major threats to the mental and physical wellness of societies [1]. Studies in war-affected populations have reported high rates of mental health problems [2]. Epidemiological research findings suggest that mental health problems are more prevalent among individuals in conflict and post-conflict zones compared to armed conflict-free zones [3]. Psychological distress is common among older people [4, 5]. It is regarded as an emotional reaction to the advert experience which is denoted by several mental illness problems, such as anxiety, depression, and bodily symptoms [5, 6]. Elderly victims in armed conflict areas are susceptible to severe stress resulting in depressive and posttraumatic stress disorders [7].

Africa is home to the largest number of victims of forced displacement and older adult internally displaced persons (IDPs) [8]. The armed conflict in Darfur, western Sudan, resulted in the destruction of hundreds of villages and the killing of thousands of civilians [9]. An extensive displacement of people occurred as a consequence of this conflict. Some sought refuge in Chad and other neighboring countries, while others found themselves clustered in camps within Darfur [10]. The war victims experienced tragic events of their relatives being killed, women being subjected to rape, and loss of their properties [9].

Despite the steadily increasing population of older IDPs worldwide, humanitarian agencies, NGOs, and governments largely ignore the needs of the elderly IDPs [7] and allocate less resources to support and serve the elderly compared to resources allocated to support children and young adults [8, 11]. More specifically, the well-being and social needs of the elderly displaced persons have been overlooked [11].

Further, mental Health literacy among refugees and internally displaced persons is generally poor. For example, among Afghani refugees and IDPs, 99.1% of the participants reported a lack of mental health knowledge [12]. In war-affected nations, stigma and mental health illiteracy remain substantial blockades to the use of adequate services. In addition, society’s beliefs about mental illness usually result in marginalizing individuals with mental illness and disheartening them from looking for professional help [13, 14]. This is additionally exacerbated by suspicion surrounding formal mental health care [15]. The lack of knowledge of mental illnesses and their treatments, in addition to stigma, represents the major barrier to the use of mental health facilities among Sudanese people [16, 17].

A systematic review of 36 studies revealed that the most vulnerable persons were old aged, women, and the widowed and that the major needs of older adults in humanitarian situations were mental health, nutrition, physical health, and personal functioning [18]. One year after the end of Lebanon’s second war, elderly people reported considerably greater levels of stress symptoms and smaller levels of posttraumatic recovery [1]. It is suggested that knowing the serious health consequences of armed conflict would help develop appropriate support systems for victims [1].

Coping refers to emotional and behavioral efforts exerted by the individual to manage a demanding event or situation [19]. There are two main categories of coping, emotion-focused and task-focused coping [20]. A further category of coping is known as avoidance coping [21]. The use of emotion-focused and task-focused coping is found to be less strongly associated with all measures of psychological distress, while the use of avoidance coping is more strongly associated with all indices of psychological distress [22]. Further, literature on gender differences in coping has shown that women tend to use more emotion-focused and avoidance coping, while men tend to use more task-focused coping [20, 23]. Further, research findings have shown that distress is negatively related to task-focused coping and positively related to emotion-focused [21, 24] and avoidance coping [21]. It has been reported that being a female and being unmarried are negatively related to mental and physical health, while increased age is strongly associated with physical ill health [25]. Moreover, elevated levels of distress were reported among women [3, 24, 26], the older, and the unmarried IDPs 3]. Studies have also revealed that increased family size in Africa is associated with less distress, depression [10, 26], social dysfunction [10], and somatic complaints [27] among IDPs.

Research conducted on psychological problems among older adult IDPs in Africa is scarce, compared to other continents [3]. Hence, there is a necessity for more research to be carried out among this population. The present study aimed at bridging the gap in knowledge in this area by investigating the effects of the armed conflict in Sudan on the mental health of older adult IDPs in Darfur and the role of coping strategies in dealing with psychological distress. Based on the literature reviewed above, the following hypotheses were made to fulfill the aims of this study: (1) High prevalence of psychological health problems will be evident among older adult IDPs; (2) Significant demographic differences in psychological health problems and coping strategies are expected; (3) Task-focused (active) coping is expected to be associated with more positive psychological health compared to emotion-focused and avoidance (passive) coping.

## Methods

### Participants

A cross-sectional design was used to gather data. One hundred nine internally displaced older adult participants were selected using a purposive sampling method. The older adult internally displaced persons were selected from the Abuzar camp around El Geneina Town. Inclusion criteria were being internally displaced victims of armed conflict aged ≥ 65 years and living in camps around El Geneina Town. Exclusion criteria were age < 65 years internally displaced victims of armed conflict, not living in camps around El Geneina Town, and victims aged ≥ 65 years who were not internally displaced. The participants’ ages ranged between 65 and 106 years (mean age = 74.93; SD = 7.07 years) the sample was drawn from a camp located around El Genena Town (Western state of Darfur). Male participants (mean age = 75.91; *SD* = 7.44) represented 64.2% of the sample while female participants (mean age = 72.92; *SD* = 5.93) represented 35.8%. About 67.6% of these internally displaced persons (IDPs) were married, 22.5% divorced, 6.9% widowed, and 2.9% single. The majority of the IDPs were from the Masaleet tribe (37.7%), followed by the Arigna tribe (31.2%), the Tama tribe (12.3%), and the rest from other tribes. Most participants received some form of formal or informal education (72.5%) while the remaining 27.5% were illiterate. They were displaced and entered the camps in 2003 through 2022.

### Measures

#### The general health questionnaire (GHQ-28) [28]

This questionnaire was factor analyzed and yielded four subscales corresponding to the original ones that measure depression, somatic symptoms, social dysfunction, and anxiety. The mean score on the scale was 9.57 (*SD* = 4.81; Alpha reliability = 0.83). The total score of the GHQ measures general distress. There are two methods of scoring the GHQ the first is the GHQ scaling method (0,0, 1, 1); the second is the Likert-like scaling method (0,1,2,3), the former is suitable for identifying non-psychotic psychiatric cases and the latter for survey research [29]. The GHQ scoring system with a cut of point of 4/5 or more is mostly used to differentiate psychiatric from non-psychiatric cases [28]. While the Likert-like scoring system is used to estimate general distress from the GHQ total score. In this study, both methods of scoring the GHQ were used. Cronbach Alpha for somatic symptoms was 0.71 (M = 4.01, SD = 1.94); for depression was 0.79 (M = 0.37, SD = 0.78); for anxiety/insomnia was 0.57 (M = 2.36, SD = 1.68) and for social dysfunction Alpha was 0.79 (M = 5.43, SD = 3.23). The GHQ-28 was used in previous studies on Darfur IDPs and was found reliable and structurally valid [9, 10].

#### The coping inventory for stressful situations (CISS) [30]

The CISS consists of 48 items assessing strategies used by individuals to deal with stressful encounters. It measures three aspects of coping strategies: avoidance, task-focused, and emotion-focused coping. There are 16 items in each dimension of coping rated on a 5-point Likert-like scale [31]. Avoidance coping entails using cognitive and behavioral efforts to avoid the problematic situation (e.g. Try to be with other people’) [32]. Task-focused involves concentrating on the problematic situation to find a solution (e.g. Work to understand the situation’; while emotion-focused, requires regulating emotions related to the problematic situation (e.g. Blame myself for procrastinating’’) [33]. Factor analysis resulted in three dimensions consistent with that of the original questionnaire. Alpha reliabilities for the dimensions were 0.88 (*M* = 34.17, *SD* = 8.55), 0.77 (*M* = 20.168, *SD* = 6.47), 0.40 (*M* = 16.09, *SD* = 3.61), respectively. The CISS scale was also used with Libyan war victims and found reliable and structurally valid [34].

### Procedures

Upon receiving approval from the Research Ethics Committee, Ajman University (Ref No: H-F-F-6-Jun-2023), permission was sought from the IDPs’ camp authorities, Humanitarian Aid Commission-Sudan. Older Adult IDPs were approached in their tents in the Abuzar camp for internally displaced persons around El Geneina Town and they were requested to take part in the study. Older adult IDPs in this camp were served by HelpAge International. The objectives of the study were explained and confidentiality of the information provided was assured. Older adult IDP participants responded to two sets of questionnaires back translated from English into Arabic earlier. Further, the participants were informed that they were free to withdraw their participation at any stage of the study. No incentives were provided to motivate participants to take part in the study. This study complies with all regulations required to conduct a study on humans.

### Data analysis

Data analyses were performed using IBM SPSS 29. Descriptive analysis was initially used to describe the demographic characteristics of the sample. Next, Pearson correlation coefficients were calculated to detect significant correlations between demographic variables, health outcome measures, and coping strategies. Additionally, t-test analysis was completed to test gender and marital status differences in general distress, anxiety, depression, somatic symptoms, social dysfunction, and coping strategies.

## Results

With the GHQ scoring method, researchers typically use a cutoff point of 4/5 [28] or 5/6 to differentiate nonpsychotic psychiatric cases from non-cases [35]. Further, 10 studies reported by Goldberg et al. used cutoff points ranging between 4 and 9 [11]. In the present study, a cutoff point of 7 was used because the authors believe that these older adult IDPs are more sensitive to symptoms and more vulnerable than other IDP groups. Therefore, using a lower cutoff point might result in false negatives which undermine older adult IDPs’ real sufferings. The use of a cutoff point of 7 resulted in 75.2% of the respondents being classified as non-psychotic psychiatric cases.

As shown in Table 1, Pearson correlation analysis revealed a significant negative association between somatic symptoms, on the one hand, and family size, and task-focus coping, on the other hand, (*r* = − 0.22, *p* < 0.05; *r* = − 0.35, *p* < 0.001, respectively). Task-focus coping was further positively related to family size (*r* = 0.36, *p* < 0.001). Those who use more task-focused coping and those with larger families reported fewer somatic symptoms. General distress and anxiety were negatively related to task-focused coping (*r* = − 0.27, *p* < 0.01; *r* = − 0.22, *p* < 0.05, respectively). The use of task-focused coping is associated with less distress and less anxiety. Social dysfunction was negatively related to avoidance-coping (*r* = − 0.22, *p* < 0.05). The more social dysfunction the less the use of avoidance coping.

**Table 1:** Correlations between the study variables

Variable	1	2	3	4	5	6	7	8	9	10
1. age	-									
2. Somatic	0.009	-								
3. Depression	0.047	0.035	-							
4. Anxiety	0.144	0.409^**^	0.367^**^	-						
5. Social Dysfunction	0.051	0.381^**^	0.294^**^	0.446^**^	-	.				
6. GHQTOT	0.085	0.722^**^	0.451^**^	0.782^**^	0.801^**^	-				
7. TASK	0.102	− 0.349^**^	0.074	− 0.215^*^	− 0.139	− 0.266^**^	-			
8. Emotion	− 0.123	0.092	0.149	0.188	− 0.125	0.074	0.014	-		
9. Avoidance	− 0.202^*^	0.035	− 0.131	− 0.014	− 0.221^*^	− 0.111	− 0.242^*^	0.100	-	
10. Family members	0.003	− 0.224^*^	− 0.013	− 0.071	− 0.031	− 0.132	0.362^*^	− 0.039	0.066	-

Note: * *p* < 0.05 ** *p* < 0.01

As shown in Table 2, two-tailed t-test analysis resulted in significant gender differences in somatic symptoms, anxiety, general distress, task-focused coping, emotion-focused coping, and avoidance. Female participants reported more distress, higher somatic and anxiety symptoms, and more emotion-focused coping (*t*(106)= -2.794, *p* < 0.01; *t*(106)= -3.634, *p* < 0.001; *t*(106)= -2.160, *p* < 0.05; *t*(106) = 2.792, *p* < 0.01; respectively) while male participants reported higher task-focused coping, and avoidance (*t*(106)= -4.429, *p* < 0.001; *t*(106) = 1.872, *p* < 0.05, respectively).

**Table 2:** Sex differences in distress, somatic symptoms, anxiety, social dysfunction, and coping strategies

Variable	Male (*n* = 70)		Female (*n* = 38)		t	df	*p*	Cohen’s d
	M	SD	M	SD				
Distress	8.84	4.77	11.39	4.06	*-*2.794	106	0.004	0.576
Somatic symptoms	3.51	1.89	4.84	1.65	-3.634	106	0.000	0.754
Depression	0.42	0.85	0.42	0.86	− 0.004	106	0.996	0.000
Anxiety	2.12	1.60	2.84	1.72	-2.160	106	0.033	0.434
Social Dysfunction	2.79	2.11	3.29	1.86	-1.236	106	0.216	0.251
Task-focused	46.30	10.46	37.42	8.93	4.429	106	0.000	0.913
Emotion-focused	32.38	7.38	37.26	10.66	2.792	106	0.006	0.532
Avoidance	22.36	5.53	20.37	4.76	1.872	106	0.032	0.386

Cohen’s d: 0.2 = small effect; 0.5 = moderate effect; 0.8 = large effect

T-test analysis revealed significant differences between the married and the unmarried (divorcees, widowed, and singles) in distress, somatic symptoms, anxiety, social dysfunction, and task-focused and avoidance coping. The unmarried participants scored higher than the married ones in all the above-mentioned dimensions, except task-focused coping and avoidance (see Table 3).

**Table 3:** Differences between married and unmarried participants in distress, somatic symptoms, anxiety, social dysfunction, and coping strategies

Variable	Married (*n* = 68)		Unmarried (*n* = 34)		t	df	*p*	Cohen’s d
	M	SD	M	SD				
Distress	8.7647	4.49583	11.4118	4.74257	-2.75	100	0.007	0.573
Somatic Symptoms	3.5588	1.90351	4.6176	1.77550	-2.71	100	0.008	0.575
Depression	0.4478	0.90927	0.4118	0.78306	0.20	99	0.844	0.042
Anxiety	2.1618	1.52208	2.8824	1.87107	-2.09	100	0.040	0.423
Social Dysfunction	2.6029	1.99357	3.5000	2.06339	-2.12	100	0.037	0.442
Task-focused	47.2059	9.53166	37.8235	9.76224	4.65	100	0.000	0.973
Emotion-focused	33.6029	8.90939	35.0294	9.13032	-0.76	100	0.451	0.158
Avoidance	22.0735	4.97551	19.8235	5.19015	2.12	100	0.036	0.443

Cohen’s d: 0.2 = small effect; 0.5 = moderate effect; 0.8 = large effect

## Discussion

Our findings suggest a high prevalence of non-psychotic psychiatric problems (75.2%) among older IDPs. These findings are consistent with the first hypothesis which postulated that a high prevalence of distress would be evident among older adult IDPs. The findings are also consistent with the previous work showing that older people in armed conflict zones are exposed to severe stress leading to depression and anxiety [36]. Further, a similar prevalence (70%) was found among Afghani refugees and IDPs [37]. However, a lower prevalence of psychological distress (49.4%) was found among Ethiopian IDPs [38]. The high prevalence of non-psychotic psychiatric disorders highlights the psychological burden borne by older victims of war and displacement experiences. These experiences may result in separation from family members, loss of traditional social roles, loss of property, and difficulty accessing the basic necessities of life.

The results of the present study generally support the second hypothesis which suggested the existence of significant demographic differences in distress and coping strategies. The negative association between age and avoidance is consistent with some studies that suggested that older adults tend to use avoidance coping strategies less frequently than younger individuals. For instance, a study by Nieto et al. [39] found that older adults were less likely to use avoidant coping strategies when dealing with stress. The results demonstrate that as individuals age, they tend to rely less on avoidance coping strategies and, instead, opt for more adaptive and proactive approaches to deal with stressful and challenging situations. This shift in coping strategies is possibly linked to improved emotional well-being and greater resilience in older IDPs.

The findings partially supported the third hypothesis that expected task-focused (active) coping to be associated with more positive psychological health compared to emotion-focused and avoidance (passive) coping. The negative association between social dysfunction and avoidance-coping is consistent with the research conducted earlier which indicated that people who employ avoidance coping strategies are more likely to experience social dysfunction [40]. This suggests that the more the social disfunction the less the use of avoidance coping. This may be due to the possibility that avoidance strategies hinder an individual’s efforts to effectively manage stress and/ or resolve the underlying problems contributing to social dysfunction.

The results of the present study suggest that task-focused coping and family size may play a role in reducing somatic symptoms. This may be attributed to the fact that task-focused coping and large families provide individuals with support and resources that may reduce the occurrence of somatic symptoms. The tendency of large family members to experience less somatic symptoms and to use more task-focused coping suggests that family size may provide some sources of social support that protect IDPs against distress, mental health problems [10], and somatic complaints [27]. Moreover, studies conducted on Sudanese refugees indicated that religious beliefs and social support, predominantly from family members and Sudanese community members, were crucial factors in coping with psychological distress [41]. Research on older adult Geeks has shown that individuals become more religious and spiritual as they advance at age [42]. Evidence has also shown that older adult African Americans increasingly depend on spirituality and religious communities (e.g. church) as coping resources [43]. This shows the significance of spirituality and religion as coping alternatives used by older adults.

The negative association between task-focused coping and somatic symptoms is consistent with early findings [44]. However, some studies found no relationship between somatic symptoms and task-focused coping [45, 46]. This result suggests that task-focused coping may divert attention away from somatic symptoms. Alternatively, older adult IDPs may suffer from some somatic problems that are difficult to manage using task-focused coping.

The association of task-focused coping with less distress and less anxiety is consistent with previous research that has found task-focused coping to be an effective strategy for reducing distress and anxiety. For example, the negative association between task-focused coping and distress is online with findings that showed lower levels of distress among those who use task-focused coping [21, 47]. The negative association between task-focused coping and anxiety is inconsistent with Orgilés et al.’s findings which showed no relationship between the two variables [48]. This inconsistency may require future studies to investigate situation-specific stress, and the nature of stressors associated with specific coping strategies. Further, the mediating/ moderating role of spirituality may be examined in future research as a study by May et al. found that Sudanese and Iraqi refugees in Australia reported religious and supernatural beliefs as causal and treatment/ coping factors in depression and post-traumatic stress [49].

The tendency of female participants to report higher anxiety symptoms, more distress, emotion-focused coping, and somatic symptoms was reported in previous studies. For instance, Hamid and Musa [9] found elevated levels of somatic symptoms among female participants. Further, Roohafza et al. [50] reported that females experienced higher levels of anxiety compared to men. The higher level of distress, and emotion-focused coping, reported by women in the present study are consistent with the results of Hamid et al. [21]. The tendency of male participants to report more use of task-focused and avoidance coping strategies is consistent with the previous findings [21, 50]. These results indicate that older adult IDP women might prefer to employ emotion-focused coping strategies to deal with anxiety and distress. Conversely, older adult IDP men might tend to utilize task-focused coping and avoidance strategies to manage their anxiety and distress. Recognizing these distinctions could assist healthcare providers in customizing their treatments and support to meet the needs of older adult male and female IDPs.

The unmarried participants scored higher than the married ones in somatic symptoms, anxiety, social dysfunction, distress, and avoidance coping, while married participants scored higher in task-focused coping. In contrast to these findings, a study by Hamid and Musa found that unmarried IDPs reported less anxiety, distress, social dysfunction, and more avoidance [9]. However, it should be noted that, unlike Hamid and Musa’s study, the sample of the present study is composed of older adult IDPs. This may explain why being single is associated with devastating effects on the older adult IDPs. Regarding Darfur culture, the elderly people are used to receiving social support from family members, single elderly may lack this type of support.

## Conclusion

This study provides valuable insights into the psychological burden faced by older adult IDPs and the potential role of coping strategies in mitigating this burden. It highlights the high prevalence of non-psychotic psychiatric problems among older adult IDPs in Darfur. The findings suggest that as individuals age, they may tend to rely less on avoidance coping strategies and, instead, opt for more adaptive and proactive approaches to deal with stressful and challenging situations. The study also highlights the importance of addressing avoidance coping strategies in individuals with social dysfunction, as a potential means of improving their social functioning and quality of life. Further, the results suggest that task-focused coping and family size may play a role in reducing the perception of somatic symptoms, with larger families providing more support and resources. Furthermore, the results suggest that older adult female IDPs may prefer to use emotion-focused coping while older adult male IDPs use more task-focused and avoidance coping in dealing with their anxiety distress and somatic symptoms. The unmarried IDPs experienced more somatic symptoms, anxiety, social dysfunction, and distress, and used less effective coping strategies (avoidance), while married IDPs used more effective coping strategies (task-focused). The findings of the present study can help healthcare providers, aid agencies, NGOs, governments, etc., customize services and support according to the unique requirements of male and female, as well as married and unmarried IDPs.

### Limitations

The present study has several limitations. First, the sample size is relatively small and self-selected, which may limit the generalizability of the findings to a broader population of older adult IDPs. Using a random sampling method and a larger sample size will enhance the outcomes of such studies. Second, the study relies on self-reported data, which may be subject to recall or social desirability biases, potentially affecting the accuracy and validity of the reported coping strategies and psychological symptoms. Combining self-reported data with data generated from interviews may increase the precision of data collected. Third, the study does not provide a comprehensive assessment of the potential cultural, social, spiritual, and economic factors that may influence the coping strategies and psychological symptoms of older adult IDPs, which may limit the understanding of the unique challenges faced by this population. The inclusion of such factors could shed more light on the coping options used by older adult IDPs. Fourth, the study does not provide a longitudinal analysis of the coping strategies and psychological symptoms of older adult IDPs, which may limit the understanding of the potential changes in coping strategies and psychological symptoms over time. Fifth, the statistical analyses used in this study do not predict the variance in distress explained by coping and demographic variables. Using more advanced statistical analysis may result in more rigorous outcomes.

### Recommendations

Future research needs to use longitudinal designs to test how coping predicts stress over time. More representative sample and fine grain analysis as well as examination of the role of religiosity, stigma, and resilience factors in experiencing and reporting psychological symptoms are recommended. Studies can also examine the possible confounding role of the demographic variables on the relationship between coping and psychological distress. It is recommended that qualified mental health professionals should be made available by governmental and non-governmental organizations to provide services related to mental health issues experienced by older adult IDPs in camps.

## Data Availability

The data that support the findings of this study are available on request from the corresponding author. The data are not publicly available due to their containing information that could compromise the privacy of research participants.

## Abbreviations

IDPs: Internally Displaced Persons
NGOs: Non-governmental Organizations
GHQ: General Health Questionnaire
CISS: Coping Inventory for Stressful Situations
